# Cystatin C Use for CKD Detection in the Veterans Health Administration System: A Qualitative Study of Barriers and Facilitators

**DOI:** 10.1016/j.xkme.2024.100830

**Published:** 2024-04-22

**Authors:** Julio A. Lamprea-Montealegre, Abigail Shapiro, Natalie A.B. Bontrager, Dena E. Rifkin, Simerjot K. Jassal, Lucile Parker Gregg, Sankar D. Navaneethan, Krista Navarra, Michael G. Shlipak, Michelle M. Estrella, Virginia Wang

**Affiliations:** 1 Department of Medicine, University of California, San Francisco, CA; 2 Kidney Health Research Collaborative, University of California, San Francisco, CA; 3 San Francisco VA Health Care System, San Francisco, CA; 4 Durham VA Health Care System, Durham, NC; 5 Center of Innovation to Accelerate Discovery and Practice Transformation, Durham VA Health Care System, Durham, NC; 6 Duke Human Vaccine Institute, Duke University School of Medicine, Durham, NC; 7 University of California, San Diego, La Jolla, CA; 8 San Diego VA Health Care System, San Diego, CA; 9 Baylor College of Medicine, Michael E. DeBakey VA Medical Center, Houston, TX; 10 Michael E. DeBakey VA Medical Center, Houston, TX; 11 Department of Population Health Sciences, Duke University, Durham, NC

**Keywords:** CKD detection, cystatin C, VHA system

## Abstract

**Rationale & Objective:**

The measurement of cystatin C has been recommended to enhance chronic kidney disease (CKD) detection and risk stratification in clinical practice. This study gathered insights into the perceptions and experiences of clinical staff regarding the use of cystatin C in CKD detection within the Veterans Health Administration (VHA) system.

**Study Design:**

A qualitative approach was employed to explore barriers and facilitators of clinical staff regarding the use of cystatin C in CKD detection within the VHA system. The Organizational Theory of Implementation Effectiveness informed the development of a semistructured interview guide.

**Setting & Participants:**

Health care providers, nurses, and clinical pharmacists from the VHA systems in San Francisco, San Diego, and Houston were interviewed between October 2021 and May 2022.

**Exposures:**

Participants' experiences with cystatin C testing.

**Outcomes:**

Perceived barriers and facilitators to cystatin C testing.

**Analytical Approach:**

Participant responses from individual interviews were analyzed by a multidisciplinary team using rapid qualitative analysis methods.

**Results:**

Fourteen in-depth interviews were conducted across the 3 VHA systems. Ten of 11 providers worked in primary care. Five key barriers to using cystatin C for CKD detection were identified. These included lack of patient awareness of CKD testing, lack of provider awareness about cystatin C, knowledge barriers about cystatin C testing, unclear roles and ownership of CKD detection, and lack of clinic support to enhance CKD detection. Suggested interventions to overcome these barriers included educational and training programs, improved clinic workflows, and electronic health record aids to support CKD detection and use of cystatin C.

**Limitations:**

The results may not be generalizable to other health care systems outside the VHA.

**Conclusions:**

The findings indicate a need for targeted interventions such as educational and training programs, improved clinical workflows, and electronic health record aids to address barriers limiting the use of cystatin C in clinical practice for enhanced CKD detection.

## Plain-Language Summary

This study assessed how clinical staff at the Veterans Health Administration (VHA) feel about using a test called cystatin C to help detect chronic kidney disease (CKD) earlier and more accurately. The research team spoke to healthcare providers, nurses, and clinical pharmacists in San Francisco, San Diego, and Houston between October 2021 and May 2022. We conducted 14 detailed interviews to understand the challenges and opportunities in using cystatin C for CKD detection. We found that participants often lacked awareness of CKD and the benefits of testing with cystatin C. There were also gaps in knowledge about how to use the test effectively, confusion over who should be responsible for CKD detection, and a need for better support within clinics to use cystatin C. To address these issues, there should be more educational programs for both staff and patients, improvements in clinic processes, and enhancements to electronic health records to better support CKD detection using cystatin C. However, the results from this study might not apply to other healthcare systems outside the VHA.

Chronic kidney disease (CKD) is an emerging global health concern, underscoring the need for timely and accurate diagnosis. However, effective and consistent diagnostic testing for CKD often falls short.^1^^,^^2^ A significant reason is the widespread undertesting of albuminuria.^3^ Additionally, estimated glomerular filtration rate (eGFR) based on creatinine might be unreliable because creatinine concentrations may be heavily affected by factors affecting its production, such as muscle mass and nutritional status.^4^

Cystatin C is less affected by external nonkidney factors than creatinine and is more strongly associated with adverse health outcomes.^5^ It provides more accurate eGFR estimates when the coefficient for race is removed.^6^ Thus, adding cystatin C to creatinine and to spot urinary albumin-creatinine ratio is now recommended to enhance CKD detection and risk stratification in at-risk populations,^4^^,^^7^ a recommendation following years of evidence supporting its accuracy and prognostic value.^5^^,^^8^^,^^9^

The adoption of cystatin C in routine CKD detection by health systems is uncertain. Traditional obstacles to CKD testing, such as the limited time and resources faced by primary care providers,^10^ could be exacerbated by specific barriers related to cystatin C. In this study, we assessed the current awareness and experience of health care professionals with cystatin C-based eGFR testing, with a focus on primary care providers. We also explored perceptions surrounding its broader implementation within the Veterans Health Administration (VHA) Health Care System (HCS).

## Methods

### Study Design and Setting

Formative evaluation of cystatin C use and perceptions of feasibility of its adoption were conducted in a qualitative study of practitioners from 3 VHA HCSs: San Francisco, San Diego, and Houston. These medical systems serve large and diverse patient populations and have had different durations of availability of cystatin C testing (Table 1).^11^ In-house capabilities for cystatin C measurements were introduced in February 2013 (San Francisco VHA HCS), January 2021 (San Diego VHA HCS), and August 2021 (Houston VHA HCS). All 3 sites comprise major teaching facilities with complexity rating “1A” denoting the highest levels of patient volume, wide availability of subspecialty services, and high involvement in teaching and research endeavors. VHA facilities report eGFR creatinine and cystatin C separately, with an option to report combined eGFR creatinine-cystatin C per local policy. Turnaround varies from hours to a day at primary centers but can extend to 2 days at remote clinics due to sample transit times.

**Table 1:** Characteristics of VHA Systems in 2020

	San Francisco VHA	Houston VHA	San Diego VHA
Patients attending primary care	21,701	79,526	51,113
Primary care providers in the main medical center	25	56	34
Cystatin C laboratory testing capabilities established	2013	2021	2021
CKD testing by comorbidity subgroups			
Type 2 diabetes; n (%)	N=4,255	N=22,437	N=9,902
eGFR creatinine	3,613 (85)	19,601 (87)	8,718 (88)
eGFR cystatin C	194 (5)	NA	NA
Hypertension without type 2 diabetes, n (%)	N=14,697	N=47,462	N=30,057
eGFR creatinine	9,877 (67.2)	36,878 (77.7)	22,513 (74.9)
eGFR cystatin C	306 (2)	NA	NA

*Note:* A VHA system encompasses one or more medical center and associated community-based outpatient clinics.

Abbreviations: CKD, chronic kidney disease, VHA, Veterans Health Administration.

### Ethical Approval

The study was approved by the institutional review boards of the University of California San Francisco (IRB number 12-29496) and the Durham VA HCS (IRBNet 1596270).

### Participants and Recruitment

We conducted semistructured interviews with primary care providers, nephrologists, and clinical pharmacists, representing relevant stakeholders involved in kidney disease detection and management. We aimed to recruit approximately 10-12 participants per site for up to 36 total key informants overall, from a mixture of personnel practicing in each local system’s main medical center and community-based outpatient clinics. Potential participants were self-identified via flyers placed in local clinics and faculty meetings and invited from snowball sampling (ie, referrals) beginning with local points of contact. Recruitment emails were sent to participants, and the study made up to 3 follow-up attempts to arrange interviews. The final sample consisted of 14 clinical staff across the 3 sites: 11 providers (8 physicians and 3 nurse practitioners), 2 nurses, and 1 clinical pharmacist. Most physicians (7 of 8) worked in primary care, and the 3 nurse practitioners worked in primary care and gerontology. Nurses focused on population health in part of their roles. The clinical pharmacist worked with nephrology and dialysis units. Participants were recruited and interviewed between October 2021 and May 2022.

### Data Collection

A multidisciplinary team developed a semistructured interview guide. Questions were developed and guided by the Organizational Theory of Implementation Effectiveness to identify and examine factors deemed important for effective cystatin C use and its implementation in VHA primary care settings.^12^^,^^13^ This framework is suitable for studying innovations that are at the stage of development at which (1) a primary adoption decision has not yet occurred at a higher level; (2) implementation requires resource allocation; and (3) coordinated use is necessary to realize benefits of the innovation. Our scope of inquiry focused on participants’ general experience in identifying CKD, their perceptions regarding the value of cystatin C testing to their scope of work and clinical practice, their perceived acceptance of cystatin C testing, and the usability and support needed for effective use of cystatin C in detection and management of CKD (Item S1, Item S2). Participant interviews were conducted by a trained qualitative analyst (AS) using VHA-approved teleconferencing services (eg, Webex and Microsoft Teams) with each interview lasting 20-30 minutes in duration (28 minutes, on average). All interviews were audio recorded and transcribed for analysis.

### Analysis

Interview data were analyzed using rapid qualitative analysis, which is an applied method used to obtain targeted findings (eg, critical factors, facilitators, and barriers) on a shorter timeline than traditional methods.^14^ This pragmatic approach follows tenants of rigorous scientific practice of reproduceable data collection and analysis. Three members of the study team (AS, NB, and VW) created a 1-page summary template for each interview using domains and main topics drawn from the interview guides and details and observations about the data collection. Summaries’ templates were completed for each interview and reviewed by a second team member. All summaries were then analyzed using matrix analysis to identify overlapping and unique responses by a priori domains of cystatin C utilization and experience with considerations of difference between sites, subsite type (eg, medical center and outpatient clinic), and participant job role. Transferability was supported with process notes, with a thick description of the analytical process, and by providing a dense description of the population studied.^15^^,^^16^

## Results

### Context for Cystatin C Use in Local VHA HCSs

Of the 14 participants, 7 were from the San Francisco VHA HCS, 5 from the Houston VHA HCS, and 2 from the San Diego VHA HCS. Participant roles included primary care providers (Medical Doctors [MDs] and nurse practitioners), a clinical pharmacist, registered nurses, and MDs in administrative leadership roles.

#### Current Practices for CKD Detection and Surveillance

Most participants reported that they routinely monitored laboratory values for CKD, especially in high-risk groups, such as older adults and patients with diabetes or hypertension or those on high-risk medications. The standard tests used were creatinine-based eGFR and urinary albumin-creatinine ratio. Providers reported consulting with nephrology for further testing guidance.

#### Awareness and Identification of Cystatin C as a Test for CKD

Six of the 14 participants independently reported the use of cystatin C for CKD testing and surveillance. No difference was observed across sites, and no nurses independently mentioned cystatin C testing.

Among those who identified cystatin C as part of current practices, it was most commonly ordered as a confirmatory test following a one-time increase in creatinine levels. Participants perceived cystatin C to be particularly useful for patients for whom elevated creatinine levels may not accurately indicate CKD, such as younger patients who use creatine supplements or exercise heavily and older patients. One participant mentioned race as a reason to use cystatin C to accurately confirm other CKD testing results.

One participant familiar with cystatin C described its perceived utility:

I think of it as a more specific and additional lab that we can use to more accurately characterize or diagnose chronic kidney disease. I know that it's expensive compared to creatinine and it's not something that I would order every time I'm checking their renal function. But I think for diagnosing and accurately characterizing where someone is it's extremely helpful.

Of those who did not independently report cystatin C use, 5 out of 8 participants reported being familiar with its use. Three participants reported not knowing about cystatin C. The clinical pharmacist participant described the utility of cystatin C, particularly in the inpatient setting. They found cystatin C to be more stable than creatinine and provided a better measure of kidney function in the inpatient setting.

#### Existing Resources and Training for Cystatin C Use

Participants’ experiences with existing resources varied widely. Most participants felt that education and training on cystatin C had been left to the individual provider. Learning about cystatin C was commonly achieved through one-to-one conversations with nephrologists or other knowledgeable colleagues, e-consults to nephrology, experience outside the VHA, and individual research via PubMed, UpToDate, and the National Kidney Foundation website. One participant found that education from nephrology consults on cystatin C and its interpretation was extremely beneficial, stating it was “very helpful to talk to an expert around a specific clinical situation.” Six participants mentioned grand rounds or other group presentations as a resource, and 1 participant mentioned learning about it from their trainees who had more recent training on CKD detection.

### Professional Experience with Cystatin C

#### Site-level Experience

In the San Francisco VHA HCS, which introduced cystatin C measurement in February 2013, cystatin C was used for most participants, largely as a confirmation of CKD in patients for whom creatinine-based eGFR may not provide an accurate measurement. However, cystatin C awareness and utilization varied. A few participants appreciated that it produces an eGFR comparable with creatinine, which aids in interpretability. One participant from the San Francisco VHA HCS knew about cystatin C but had never personally ordered the test; 2 knew about cystatin C but relied on other providers to order it, and 1 had never heard of cystatin C.

San Diego VHA HCS implemented cystatin C testing in January 2021. Both interview respondents frequently used cystatin C as a confirmatory measure of CKD.

Houston VHA HCS capabilities for cystatin C use began in August 2021, and 3 respondents reported using cystatin C, one of whom had only recently integrated the test into their workflow. All of them found cystatin C to be an important addition to CKD detection and management, particularly for patients for whom creatinine-based eGFR may not be accurate. Two participants were not aware of cystatin C, one of whom stated that the test was not available at their location.

#### Ownership of the CKD Management Process

Several participants reported uncertainty about who in their clinic “owned” the CKD management process, with multiple suggesting that it fell to the primary care provider. Some mentioned the roles of population health nurses and clinical pharmacists who manage CKD alerts on team panels. Three participants identified a specific physician as the clinical champion of CKD in their clinic.

### Limitations of Current CKD Detection and Management and Use of Cystatin C

#### Patient-level Barriers

Two providers identified challenges with patients’ lack of awareness about the purpose of laboratory tests, particularly urine testing; they shared their patients’ misconceptions that all urine testing is intended for illegal drug screening. Another participant noted patient issues with transportation in their rural area and highlighted patient-reported issues with limited scheduling availability at their local clinical laboratory.

#### Provider-level Barriers

Many participants cited workload and staffing issues as significant limitations for cystatin C use. The concerns included a lack of providers, long working hours, instability among providers at a site, and the need for better training and guidance on CKD testing for the trainees in their clinic. Other respondents noted a broader awareness and education gap about cystatin C, including what it is, how it is used, and how to interpret its results.

A lack of clarity around roles and ownership of CKD identification was mentioned by several respondents. Nurses reported relying on primary care providers to choose laboratory orders. Primary care providers relied on specialists for consults and to order additional testing, whereas specialists relied on primary care providers to manage CKD earlier in the disease process. One participant described their clinic’s care provision as siloed, with providers failing to communicate with each other about best practices.

#### System-level Barriers

Participants noted challenges due to a lack of education and automated processes to maintain awareness of CKD and to assist them in managing CKD with their patients. This issue was especially significant given the context of limited staff availability and education/training about cystatin C. Participants noted the absence of the following elements that might enhance a system’s ability to improve identification and surveillance of CKD: reminders or dashboards for renal testing, alerts in the electronic medical record, education guidance that accompany laboratory results for interpretation, or performance measures.

### Potential Facilitators to Optimize Cystatin C Use for CKD Detection

#### Resources and Trainings to Support Cystatin C Use

Participants made a wide range of suggestions for training and educational resources (Table 2). These included a general introductory background on cystatin C (eg, what it is, how it originated, and why it is useful). Participants commonly wanted education on how to interpret the results and act on them in the clinical setting. Many also specifically requested clinical decision guidance for cystatin C.

**Table 2:** Suggested Resources and Training to Support Cystatin C Use

Topics	Modality of Resources, Trainings
• Clinical decision guidance • Interpreting results • Background information • Updated research	Expert consultations
	• CKD e-consult mechanism
	• Short presentations to clinics by guest speakers
	• Clinical champion or "go-to person" within their clinic
	Embedded guidance within the EHR
	• Clinical reminders and alerts
	• Reflexive laboratory orders
	• Clinical decision guidance
	• Interpretation of results
	• Inclusion on a CKD dashboard
	• Inclusion on a clinical or laboratory performance dashboard
	Ad hoc resources
	• "One-pager" documents with basic information on cystatin C
	• Emails to primary care service from nephrology service about cystatin C with up-to-date research and best practices
	• Access to top-tier nephrology journals for independent research
	• Brief, asynchronous online training through VHA educational portal

Abbreviation: CKD, chronic kidney disease, EHR, electronic health record; VHA, Veterans Health Administration.

Other recommendations focused on the operational logistics of cystatin C in clinics and varied widely by specific content and delivery method. Some participants, particularly those from community-based outpatient clinics, desired a specialty CKD e-consult mechanism to have as-needed training by specialists for relevant cases.

Participants wanted guidance embedded within the electronic medical record system to direct their usage of cystatin C. These could take the form of clinical reminders, alerts, reflexive laboratory orders for appropriate cases, and other tips for using cystatin C as “just in time” education. A few participants mentioned wanting to include cystatin C on a CKD dashboard, or on a clinical performance dashboard. Several participants wanted short “one-pager” documents that they could easily use for guidance. A few participants wanted ongoing education via reminders or nephrology emails with updated information about cystatin C.

Two participants mentioned wanting greater access to top-tier nephrology journals through the VHA, which are currently inaccessible. One participant wanted brief, asynchronous online training to be required of all relevant providers. A few participants mentioned wanting a local point of contact or clinical champion with some expertise within their clinic.

Respondents at community-based clinics desired short presentations from guest speakers with expertise in cystatin C.

#### Workflow Changes for Cystatin C

Participants proposed several workflow changes for cystatin C use across several domains to support cystatin C use (Table 3). The most common suggestion was automation of processes; 3 participants wanted cystatin C orders to be reflexive for cases in which criteria were met. Two participants suggested creating separate renal panels, one including cystatin C for patients for whom cystatin C is “automatically warranted” or likely to be the more accurate measure. One participant said:

If there were something automatic like that to be able to allow the clinician to say ‘Yes, I want to order the test’ and then it comes up and you’re done. You order the test, it’s done, or ‘No’ if you don’t, then you just move on. That way it takes the guesswork out of it.

**Table 3:** Suggested Workflow Changes and Personnel Involvement

All	Nurses	Primary Care Providers	Laboratory Staff
EHR format/function • Reflexive orders • Separate renal panels, with one including cystatin C EHR tools • CKD dashboard with integrated education and clinical support • Performance metrics • eGFR calculator for creatinine and cystatin C	EHR permissions • Clinical reminders that nurses can address • Add cystatin C to standing laboratory orders that nurses can request Research/process improvement • Testing cystatin C implementation in clinics	Research/process improvement • Testing cystatin C implementation in clinics • Identify clinician champion of CKD	Process improvement • Decreased laboratory turnaround time

Pharmacists	Nephrologists	Administration/Leadership	Education Service
Role changes • Increased involvement of clinical pharmacists in CKD management	Resources and training • Educate on cystatin C at primary care team meetings • Provide cystatin C specific e-consults	Prioritization • Championing cystatin C and CKD management	Resources and training • Develop and disseminate trainings on cystatin C in the VHA educational platform

Abbreviations: CKD, chronic kidney disease; eGFR, estimated glomerular filtration rate; EHR, electronic health record; VHA, Veterans Health Administration.

Six participants wanted support to align cystatin C with their workflows within the electronic medical records. Two respondents suggested clinical reminders that nurses would address, 2 wanted a CKD dashboard with integrated education and clinical support, and 1 wanted performance metrics to aid their use of cystatin C. One participant also wanted an easy-to-access calculator to consider both creatinine and cystatin C eGFRs to determine kidney function.

A few participants proposed enabling nurses to order cystatin C, as they do for other renal tests.

Two participants wanted to decrease turnaround time for cystatin C laboratory testing; a participant working in a community-based clinic described waiting over 2 weeks for results due to delays in sample processing time.

#### Personnel Involvement in CKD Identification, Surveillance, and Management

Perceptions of provider involvement in CKD management varied considerably. Many participants reported the need for CKD detection, surveillance, and management to be championed by leadership at the clinic, department, and medical center levels. Some participants believed that assuming this responsibility should be at the discretion of individual primary care providers within their teams. One participant stated, “the person who has the biggest interest in doing it … regardless of who they are.” Another participant suggested that a person keen on quality improvement could employ a Rapid Process Improvement Workshop project to determine the optimal implementation of cystatin C in their clinic.

Five participants suggested that nurses could lead population-level management of CKD. By increasing nurse involvement in laboratory orders and surveillance of cystatin C laboratory tests that can be placed into standing orders, nurses could oversee cystatin C use and CKD detection and management. Such a system would also allow for greater involvement of clinical pharmacists, as desired by 2 other participants.

Participants acknowledged variation in resource availability across VHAs, noting that the decisions about who to involve is highly dependent on clinic site and relies on the person who has the right expertise or interest. Several respondents described that joint ownership of these processes (eg, collaboration) would generally fall to personnel from primary care teams, hospital medicine, and nephrology. One participant felt that the educational service line should also be involved to develop and disseminate training on the topic through the VHA educational portal.

## Discussion

This qualitative study primarily captures the perspectives of primary care providers and other clinical staff on the use of cystatin C in the VHA for CKD detection and monitoring. The findings show considerable variation in the understanding, usage, and perceived value of cystatin C among participants. The use of cystatin C is limited due to lack of awareness, gaps in knowledge and training, and workflow challenges. These findings suggest several opportunities to improve CKD detection and management within the VHA, with potential implications for other health systems.

The barriers to the use of cystatin C identified in our study were multifaceted and involved barriers specific to cystatin C and challenges to optimal CKD detection. Generally, several providers admitted to a knowledge gap about cystatin C, its use, and interpretation. These results emphasized the need for increased provider education and for the implementation of training programs related to the use and value of cystatin C in CKD detection. While these barriers may stem from the relative novelty of cystatin C, a challenge for health systems will be how to integrate cystatin C testing with creatinine and urinary albumin-creatinine ratio in a manner that does not add to the already high provider workload.

In addition to provider-level barriers, participants noted a lack of patient awareness and understanding of CKD and its diagnostic tests, which are critical to CKD testing. This is consistent with research that shows that <25% of patients are aware of having CKD.^1^ Improving awareness of CKD is crucial for ensuring patient cooperation, adherence to testing recommendations, and acceptance of initiation of disease-modifying treatments. System-level limitations included the lack of automated processes to maintain CKD awareness, a gap in training resources, and the absence of specific ownership of CKD identification and management within clinics. These findings align with prior research that has identified divergent perceptions of primary care providers and specialists regarding roles in the management of patients with CKD and the need for better care coordination.^17^

The potential solutions suggested by participants highlighted the importance of integrating cystatin C use into existing workflows and the electronic medical record. The automation of cystatin C orders and implementation of reminders and renal panels inclusive of cystatin C could potentially address many of the reported challenges. Moreover, improving access to relevant training resources and creating specialty CKD e-consult mechanisms could help improve provider self-efficacy in CKD detection and management. Participants also advocated for the establishment of a clear ownership structure for CKD management, suggesting a collaborative approach involving primary care teams, nephrology, hospital medicine, pharmacy, and nursing.

Despite the novel insights gained from our study, we acknowledge its limitations. This research was conducted within the VHA system, which may limit the generalizability of the findings to other health systems due to the availability and cost of cystatin C and potential differences in structure, staffing, resources, and patient demographics. For example, the cost for cystatin c is approximately $5-$10 USD per assay, which may be different than in other health systems. However, the VHA is the largest integrated HCS in the United States, and the challenges and opportunities identified herein may be relevant to other health systems as well. In addition, we necessarily reduced our sample size due to the larger historical conditions of our data collection during the winter 2021-2022 wave of the COVID-19 pandemic, during which the health care providers we sought to enroll had limited availability for participation because of high workloads and overburdened clinical needs. The modest sample size precludes discerning role-specific or site-based differences. Nevertheless, this research illuminates mainly general practitioner perceptions from 3 sites, each with varying durations of exposure to cystatin C testing availability. Additional research should extend our work by primarily including nonprimary care providers working in different locations and health systems.

Enhancing early detection and management of CKD is a public health priority. This study underscores the necessity for a comprehensive approach that engages patients, improves provider education and training, and streamlines workflows to optimize the use of cystatin C in CKD detection and management. Future research should further explore the implementation and effectiveness of these strategies across different settings, aiding the continuous efforts to improve CKD outcomes.

## Supplementary Material

Supplementary File (PDF) Item S1-S2.
