# Supplementary material for: Cystatin C Use for CKD Detection in the Veterans Health Administration System: A Qualitative Study of Barriers and Facilitators
Source: Kidney Med. 2024 Apr 22;6(6):100830. doi: 10.1016/j.xkme.2024.100830 (PMC11127258; doi:10.1016/j.xkme.2024.100830)
Supplement: Supplementary File (PDF) — Item S1-S2. [file mmc1.pdf]

Item S1: Quotes by Theme

Domain and Theme	Quote
Context for Cystatin C Use in local VA Health Care Systems	
Current practices for CKD detection and surveillance	"Well, I don't think we have a specific test. They come in for their annuals and we're looking at a complete metabolic panel. And … you're looking at creatinine and GFR particularly. I'm sensitive if the patient is a diabetic … I want their sugar under control because I understand the relationship to diabetes and CKD, but as far as one specific marker or one specific group of testing, I don't believe we have that. Or if we do, I'm not aware of it. I'll say it like that."
Awareness and identification of cystatin C as a test for CKD	"[Cystatin C testing] came to our Medical Center last summer, but I will admit to my ignorance, that I did not realize that until like a week and a half ago. No, I was listening to a grand rounds that they announced it was now available. So now that we know it's here, I think it's something we definitely will start using. There's good evidence for using it, but I simply didn't realize it was something we could readily get."
Existing Resources and Training for Cystatin C Use	"A nephrologist spoke to our primary care group here in [VAMC]. That was probably 6-8 months ago. That's really the only formal education I think we've had as a primary care clinic. I use available resources online frequently, such as UpToDate. And other quick measures and and quick items that will help with a 2-3 minute education because I don't have a lot of time to to read in-depth articles. And so usually it's a review article that's been prepared, and I've looked at 1 or 2 review articles. But I must admit that total time I probably spent reading about it is less than 10 minutes."
Professional Experience with Cystatin C	
Site-level Experience	"I think that there's sort of an awareness of [Cystatin C] at the VA Medical Center because of the work of researchers like [Name]. I think that it does actually get used in our setting a fair amount and a lot of the residents I work with for example, are aware of it and it and … when I'm precepting will bring it up in specific contexts like 'oh this person's GFR is reduced but I think it might be because of you know XYZ, like muscle mass or age or something like that.' So they'll propose ordering Cystatin C. And it's pretty easy to order in our system."
Ownership of the CKD Management Process	"No, there's nobody who's a champion for this is as far as I know in the [VAMC]. I'm not aware that there are any CKD champions that are typically designated."

Limitations of Current CKD Detection and Management and Use of Cystatin C	
Patient-level barriers	"Mostly I'm able to get people in to get their renal testing done. But for some patients, they have transportation issues or they might you know just live not very close to the clinic and they don't want to drive in just just for lab, so I'm just making sure they understand the importance and then really transportation I would say is the biggest obstacle for them. … and so is getting them scheduled. Sometimes it's problematic, and like at the main Medical Center they can just walk in to get labs anytime, but at the CBOCs [community-based outpatient clinics], most of them require them to be scheduled."
Provider-level barriers	"I think for for me … it's one of those things like, you only really know your own practice. It's not something I'm discussing with other providers. So maybe other providers feel like they have a much better understanding of it, but I think in this case, where it's really quite dramatic and could change treatment options, when it's that big of a difference, it feels hard for me to know how to interpret that for this patient and and if I really should just be relying on the cystatin C versus the creatinine for this older man … And so, you know, do I think about him as having a EGFR of [Stage 2 CKD level], which is what his creatinine would suggest, or an EGFR of [Stage 3b CKD level], which is what his Cystatin C would suggest, which are just so dramatically different. And then, if I do really think of him as having more significant CKD then? How do we make that clear for other providers in his chart and if they are not used to thinking about cystatin C."
System-level Barriers	"As Pact RNs we actually don't see the clinical reminder of whether a patient is due for labs. There's no flags or anything like that that tells us this patient is a CKD patient and we should be mindful of that. It's more of a collaboration with the provider, the PCP. We do a lot of performance measures or population management."

Potential Facilitators to Optimize Cystatin C Use for CKD Detection	
Resources and Trainings to Support Cystatin C Use	"Sometimes [in the EMR lab order pages] there's little tips like, if they think it should only be ordered once a year, or in the diagnosis of CKD, or if there's a change in the creatinine. Then some of those tips can be just embedded there so that in the workflow of a PCP they can get that in like just-in-time sort of education when ordering."
Workflow Changes for Cystatin C	"…incorporate something into the EMR, I'm not sure how you would provide a decision support. Like say, if you have an alert that you could use. Maybe if you have an abnormal GFR result maybe you could put a little alert in CPRS, saying 'this person 's GFR is below 60, Do you want to order a confirmatory test?' And then automatically orders the cystatin C …Then it would be up to the clinician to decide if they want to order that test or not."
Personnel Involvement in CKD Identification, Surveillance, and Management	"I think that's the PCP, the clinical pharmacist, and the RN who who would be working collaboratively to do that, and their clinical pharmacist- mainly because they're seeing and helping to manage our patients with hypertension and diabetes, which are the main risk factors for CKD in our country. And then our PACT RN, who really owns a lot of our panel management and population health efforts together with the PCP, but could execute very well if we were able to generate a list of patients and a protocol that we wanted them to to follow. And then of course, the PCP just ongoing and natural clinical visits and with panel management. So I think those 3 are the key players. And then if our facility was like, 'we want to find every last person,' or help in the awareness of it … I think you're probably reaching out to people from the lab as well."

Item S2: C Cubed Provider Interview

Study ID:

Interviewer:

Date:

Note to IRB: As this is a semi-structured qualitative interview, exact question order and wording may vary.

1. To get started, I'd like you to walk me through what CKD identification and surveillance looks like in your clinic at [VAMC name].
   Probes:
   a. How do you usually test for CKD?
   b. In what ways is testing for CKD important?
      i. For whom?
      ii. Has this changed over time?
         1. What prompted these changes (e.g. new Tx like SGLT-2 inhibitors?)
   c. How is this information being used?
   d. How have these practices changed over time?
   e. What limitations do you perceive about the current practices?
   f. Who 'owns' CKD identification and surveillance in your clinic? Who is involved at each step of the process?

Now I'd like to talk about one aspect of CKD care that you [did/didn't] mention, checking Cystatin C for CKD identification and management.

2. I'd like to learn what your experience is with Cystatin C-based eGFR and the role it plays in CKD identification and surveillance at your site. Have you used Cystatin C? Could you tell me about how you use it?

   [*If participant does not know what Cystatin C is/is used for, read the following: Cystatin C is a biomarker used in an equation with sex and age to calculate an estimated Glomerular Filtration Rate (eGFR). Cystatin C is particularly useful for estimating kidney function when creatinine production is variable or unpredictable. Your site has introduced Cystatin C to enhance CKD detection and stratification in primary care.]*
   Probes:

   a. When do you use it? For whom?
   b. Who makes the decision to request/use Cys C labs?
   c. Who is involved at each step?
   d. How is this information being used?
   e. What limitations do you perceive about the current practices?
   f. In what ways, if any, is the use of Cys C helpful for you in CKD identification and surveillance?
   g. Is there someone you could identify as a clinical champion of Cystatin C?

3. Cystatin C testing is fairly new at your site; could you tell me a bit about what resources or training you've used to learn about it?
   Probes:
   a. Availability/ordering of lab testing?
   b. Education on interpreting results?
   c. Decision support or education on integrating results into clinical management?

Next, I'd like to discuss ways the VA can optimize the use of Cys C in the primary care clinic.

4. What might you change to make the support and education materials more useful to you?
   Probes:
   a. Education materials (content or method)
   b. Guidelines
   c. Clinical support
   d. Dashboard

5. What might you change about the workflows around Cys C at your site?
   Probes:
   a. Identifying risk factors for follow-up diagnostic testing/monitoring
   b. Ordering labs
   c. Receiving lab results: reporting and interpretation
   d. Translating lab results → clinical decision-making
   e. Following through on clinical decision-making (e.g, coordinating with patient to get lab testing done, communicating results to patients)

6. The VA is interested in rolling this out to more sites going forward. Do you have any thoughts about who in Primary Care should be involved/responsible for CKD identification, surveillance, and management?
   Probes:
   a. All clinicians (MD, NPs, PAs)
   b. Case manager
   c. Clinical pharmacist
   d. Nephrologists (especially…what do you think nephrologist's role should be in cases of early CKD?)

7. Is there anything else you'd like to add about using Cystatin-C for identification or surveillance of CKD in your clinic, or the supports your clinic might need to integrate it better?

Thank you – those are all my questions. I really appreciate your time and contributions today.

8. Is there anyone else you would recommend we speak to next?
